# Deletion of SNAP-23 Results in Pre-Implantation Embryonic Lethality in Mice

**DOI:** 10.1371/journal.pone.0018444

**Published:** 2011-03-29

**Authors:** Young Ho Suh, Aki Yoshimoto-Furusawa, Karis A. Weih, Lino Tessarollo, Katherine W. Roche, Susan Mackem, Paul A. Roche

**Affiliations:** 1 Experimental Immunology Branch, National Cancer Institute, National Institutes of Health, Bethesda, Maryland, United States of America; 2 Receptor Biology Section, National Institute of Neurological Disorders and Stroke, National Institutes of Health, Bethesda, Maryland, United States of America; 3 Neuroscience Graduate Program, Department of Pharmacology, Ajou University School of Medicine, Suwon, South Korea; 4 Mouse Cancer Genetics Program, Center for Cancer Research (CCR), NCI-Frederick, National Institutes of Health, Frederick, Maryland, United States of America; 5 Cancer and Developmental Biology Laboratory, Center for Cancer Research (CCR), NCI-Frederick, National Institutes of Health, Frederick, Maryland, United States of America; Sanford-Burnham Medical Research Institute, United States of America

## Abstract

SNARE-mediated membrane fusion is a pivotal event for a wide-variety of biological processes. SNAP-25, a neuron-specific SNARE protein, has been well-characterized and mouse embryos lacking *Snap25* are viable. However, the phenotype of mice lacking SNAP-23, the ubiquitously expressed SNAP-25 homolog, remains unknown. To reveal the importance of SNAP-23 function in mouse development, we generated *Snap23*-null mice by homologous recombination. We were unable to obtain newborn SNAP-23-deficient mice, and analysis of pre-implantation embryos from *Snap23* ^Δ/wt^ matings revealed that *Snap23*-null blastocysts were dying prior to implantation at embryonic day E3.5. Thus these data reveal a critical role for SNAP-23 during embryogenesis.

## Introduction

Vesicle-mediated intracellular protein trafficking is essential for a wide variety of cellular processes including both constitutive protein transport and regulated exocytosis. The protein machinery regulating vesicle trafficking is conserved in organisms ranging from yeast to human, and among them the SNARE (soluble N-ethylmaleimide-sensitive factor attachment protein receptor) complex has emerged as specialized machinery in mediating vesicle-target membrane fusion [1]. Although there are many factors that interact with and modify the SNARE complex, the trimeric core complex of syntaxin, VAMP/synaptobrevin, and SNAP-25 are the prototypical components of the SNARE complex and together play a key role in membrane fusion process [2].

Since membrane-membrane fusion events are critical for all cell types and are important for maintaining the orderly movement of cargo proteins from one intracellular compartment to another, it is not surprising that there are a wide variety of distinct SNARE isoforms that reside on distinct intracellular compartments, thereby ensuring appropriate homotypic and heterotypic membrane fusion events. For example, there are a wide variety of syntaxin and VAMP isoforms in eukaryotic cells that are expressed on particular organelles in essentially all cell types. By contrast, SNAP-25 is only expressed in neuronal/neuroendocrine cells and the role of SNAP-25 in the SNARE complex in non-neuronal tissues is taken-over by the related protein SNAP-23 [3]. SNAP-23 is ubiquitiously expressed and has been shown to play a role in diverse protein trafficking events including GLUT4 transport in adipocytes [4], mast cell degranulation [5]–[7], dense core granule release in platelets [8], cholecystokinin-regulated exocytosis in pancreatic acinar cells [9], and surface expression/recycling of transferrin receptors [10], the glutamate transporter EAAC1 [11], and NMDA receptors [12], [13].

Genetic ablation of various syntaxin and VAMP isoforms does not significantly impair embryonic development, revealing the importance of genetic redundancy of SNARE function in development. Surprisingly, deletion of SNAP-25 does not affect embryo viability, although *Snap25*-null mice die at birth due to neuromuscular abnormalities [14]. By contrast, the importance of SNAP-23 in mouse development and embryonic viability remains unknown. We now report that deletion of *Snap23* results in pre-implantation embryonic lethality, highlighting the importance of this ubiquitous SNARE in mouse development.

## Results and Discussion

### Generation of SNAP-23-deficient mice

**Figure 1:**
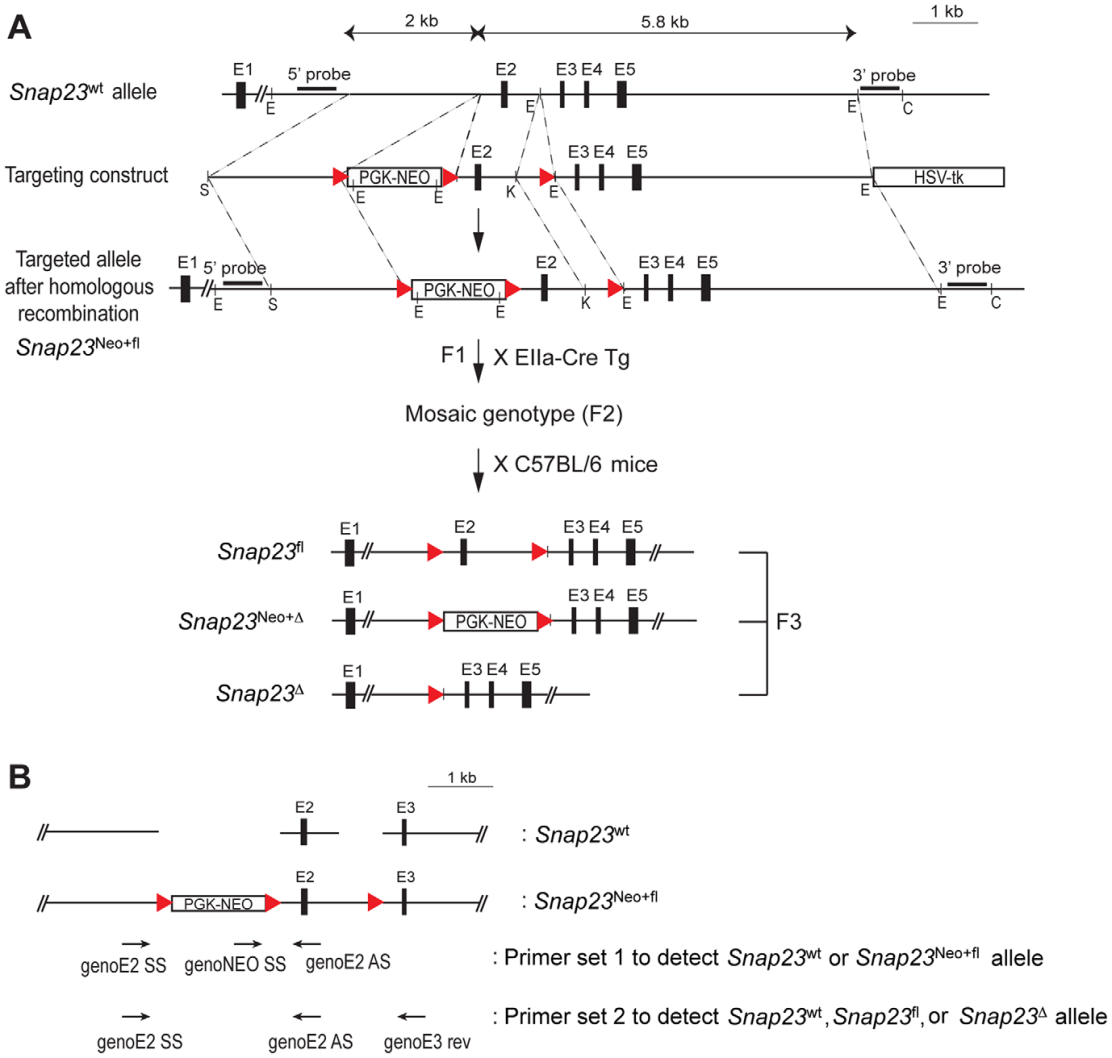
Gene targeting strategy for the generation of *Snap23* ^fl/wt^ and *Snap* ^Δ23/wt^ mice. (**A**)Schematic representation of the genomic structure of wild-type (wt) and targeted alleles of *Snap23*. Homologous recombination with the targeting construct inserts a neomycin-resistance gene (PKG-NEO) and exon 2 (E2) flanked by three loxP sites represented as triangles. HSK-tk was used for negative selection. Exon numbers with relative position, 5′- and 3′- flanking Southern blotting, and a partial restriction map are indicated. Restriction sites: C, ClaI; E, EcoRI; K, KpnI; S, SpeI. Female *Snap23* ^Neo+fl/wt^ heterozygous mouse harboring the targeted allele (F1) was bred with male EIIa-Cre transgenic mice. Offspring harboring mosaic alleles (F2) through partial and/or total excision of loxP-flanked sequences were generated. EIIa-Cre^+^ F2 mosaic male mice were further bred with C57BL/6 female mice to separate the *Snap23* ^fl/wt^, *Snap23* ^Neo+fl/wt^, and *Snap23* ^Δ/wt^ alleles as shown (F3). (**B**) Location of oligonucleotide primers used to discriminate mice harboring different *Snap23* alleles following Cre-mediated excision. Genotyping for the *Snap23* ^wt^ and *Snap23* ^Neo+fl^ alleles was performed by PCR Primer set 1 and yielded fragments of 266 bp and 400 bp from the *Snap23* ^wt^ and *Snap23* ^Neo+fl^ alleles, respectively. The Neo- *Snap23* ^fl^ and *Snap23* ^Δ^ alleles were identified by PCR using PCR Primer set 2, which yields fragments of 266 bp, 400 bp, and 492 bp from the Neo- *Snap23* ^wt^, *Snap23* ^fl^, and *Snap23* ^Δ^ alleles, respectively (also see Table 1).

**Figure 2:**
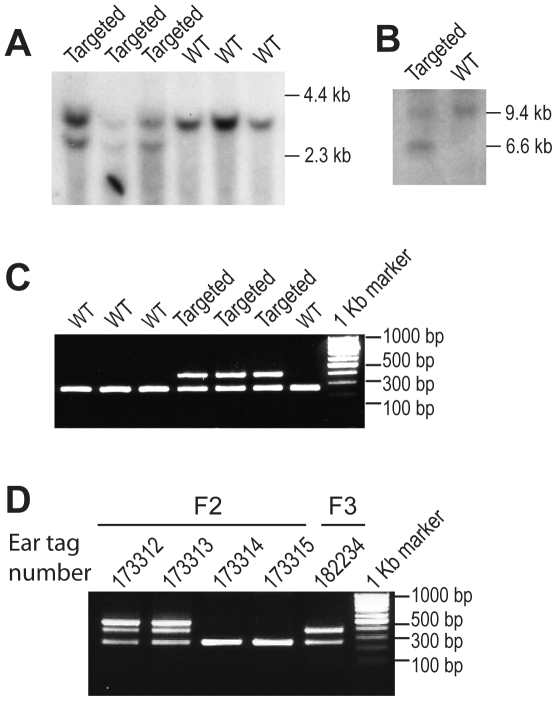
Generation of mice harboring the *Snap23* ^fl^ or *Snap23* ^Δ^ allele. (**A and B**) Southern blot analysis of genomic DNA from targeted *Snap23* ^Neo+fl/wt^ ES cell clones. (**A**) EcoRI-digested genomic DNA was hybridized with a 5′ probe as shown in Figure 1A. Southern blot analysis of EcoRI-digested genomic DNA using the 5′ probe revealed a 3.4 kb or 2.5 kb fragment in the *Snap23* ^wt^ or *Snap23* ^Neo+fl^ targeted allele, respectively. (**B**) Southern blot analysis of ClaI/KpnI-digested genomic DNA using a 3′ probe revealed a 9.6 kb or 6 kb fragment from the *Snap23* ^wt^ or *Snap23* ^Neo+fl^ targeted allele, respectively. (**C**) Genomic PCR from *Snap23* ^Neo+fl/wt^ heterozygous mice using PCR Primer set 1. The expected size of PCR products is 266 bp from the *Snap23* ^wt^ allele and 400 bp for the *Snap23* ^Neo+fl^ allele. (**D**) Genotyping was performed using PCR Primer set 2 to identify *Snap23* mosaic mice. Three PCR products of 266 bp, 400 bp, and 492 bp were obtained from mice with ear tag numbers 173312 or 173313, indicating these mice harbor a mixed mosaic genotype (F2) depicted in Figure 1A. Mice with ear tag number 173314 or 173315 yielded a single PCR fragment of 266 bp, indicating these possess either the *Snap23* ^wt^ or *Snap23* ^Neo+Δ^ allele. The mosaic EIIa-Cre^+^ male mouse 173312 was mated with a female C57BL/6 mouse and one of the pups (F3; ear tag number 182234) was found to be EIIa-Cre^-^ and possessed the *Snap23* ^fl^ allele. Genomic PCR from this mouse revealed only two PCR fragments corresponding the *Snap23* ^wt^ and *Snap*23^fl^ allele, indicating that this was a *Snap23* floxed exon 2 heterozygous mouse.

We generated *Snap23-*deficient mice using a conventional gene replacement targeting method through homologous recombination [15]. A targeting vector was designed to delete *Snap23* exon 2, which is the first coding exon of the mouse *Snap23* gene [16], by Cre-mediated excision (Figure 1A) and PCR Primer sets were designed to screen for wild-type and targeted *Snap23* alleles (Figure 1B). A neomycin-resistance gene under the control of the PGK promoter (PGK-Neo) was placed in front of exon 2 and PGK-Neo, as well as *Snap23* exon 2, were flanked by loxP sequences. The linearized targeting vector was introduced into the CJ7 ES cells and G418/FIAU-resistant clones were screened for homologous recombination at the *Snap23* locus. Southern blotting of EcoRI-digested genomic ES cell DNA with a 5′ probe revealed a 3.5 kb fragment from the wild-type *Snap23* gene as well as a 2.5 kb fragment that was the product of homologous recombination at the 5′ end of exon 2 (Figure 2A). Similarly, when ClaI/KpnI-digested DNA was hybridized with a 3′ probe we observed a 9.4 kb wild-type fragment as well as a 6.0 kb fragment in targeted ES cells (Figure 2B). Of 85 neomycin-resistant/ganciclovir-sensitive ES cell clones analyzed by Southern blotting, seven clones had undergone homologous recombination in both the short-arm (5′) and long arm (3′) sequences of our targeting construct. This Southern blotting result was confirmed using a PCR-based genotyping assay (Figure 1B, Figure 2C, Table 1). Four of these targeted ES cell clones were then used to generate chimeric *Snap23* ^Neo+fl/wt^ founder mice.

**Table 1:** Oligonucleotide primers used in this study and estimated size of PCR products.

Name of primers	Sequences (5′ to 3')
genoE2 SS	TGCCCATAGGTTGTCAGACT
genoNEO SS	TCACCTTAATATGCGAAGTGG
genoE2 AS	ATGTGCTAACCATGACCTTGA
genoE3 rev	GAGAGACCTCAGATGGTGGAG
CreSS	CCGGGCTGCCACGACCAA
CreAS	GGCGCGGCAACACCATTTTT
SA-4400SpeI	ACTAGTTGCTTCACCTCTTCAAAGTTTC
SA-6400SalI	GTCGACTTTCAGCCTGTACATCCTGTGC
E2-6400SpeI	ACTAGTGATCAGAAGCTCAAGGTCATGG
E2-7308KpnI	GGTACCCTTCCAGAATTGCAGGTAACTG
E2-5probeSS	TGCCCAGAACTACTGTAAAGC
E2-5probeAS	TGCTGTTTAAAGCATCTCTGC
E2-3probeSS	GGGTAGAGCAATGGGTGTATT
E2-3probeAS	AGAATGCACGTCGTCTTGTAG

Name of primers	Estimated size of PCR products
genoE2 SSgenoNEO SSgenoE2 AS	WT allele: 266 bpNEO**^+^** floxed allele: 400 bp
genoE2 SSgenoE2 ASgenoE3 rev	WT allele: 266 bpNEO**^–^** floxed allele: 400 bpNEO**^–^** exon2**^Δ^** allele: 492 bp
CreSSCreAS	Cre**^+^** allele: 444 bp

The oligonucleotide sequences used in this study are listed in the upper table. The oligonucleotide primers were used to genotype the mice, to obtain genomic fragments of *Snap23*, and to generate probes for Southern blot analysis. The estimated sizes of PCR products obtained during genotyping the mice are indicated in the lower table.

To generate *Snap23* exon 2 floxed mice as well as mice in which *Snap23* exon 2 was deleted, *Snap23* ^Neo+fl/wt^ mice were mated with transgenic mice expressing Cre under the control of the EIIa promoter. The adenovirus EIIa early promoter is known to be transcriptionally activate in mouse oocytes and early embryos prior to implantation in the uterus [17]. Of note, when male EIIa-Cre transgenic mice are mated with female mice harboring multiple loxP sites, partial Cre-mediated excision can occur among different loxP sites, leading to the production of mice with a mosaic genotype [18]. This mosaic (F2) genotype can be observed by PCR analysis of genomic DNA (Figure 2D) and can be segregated into the discrete recombinant alleles in the next (F3) generation by mating with wild-type mice [18]. This breeding gave rise to *Snap23* ^fl/wt^ and *Snap2*3^Neo+Δ/wt^ mice (Figure 1A, Figure 2D). Genomic PCR from F3 mouse tail DNA identified a mouse (number 182234) that contained only the *Snap23* ^wt^ and *Snap23* ^fl^ alleles (Figure 2D), demonstrating that the mosaic alleles were segregated individually in this mouse.

**Figure 3:**
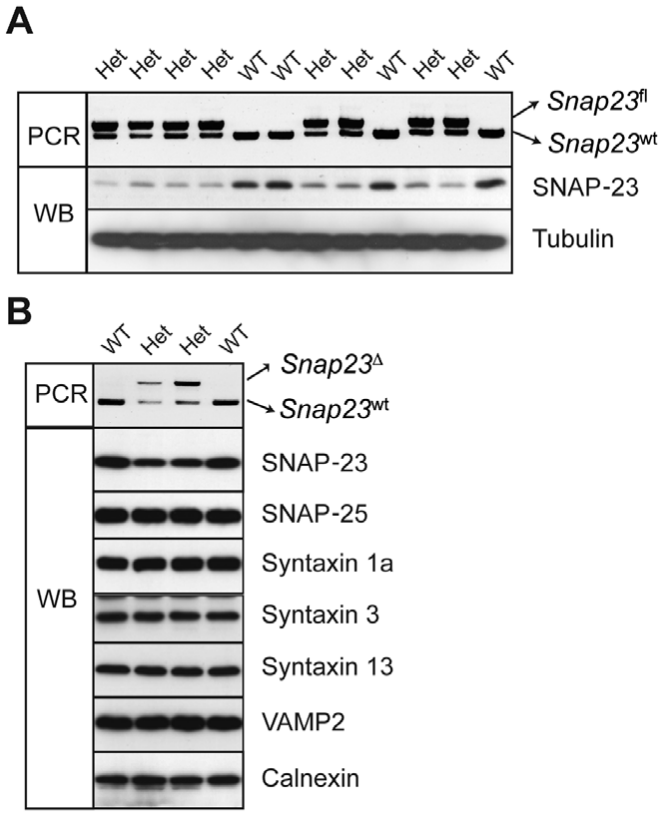
Expression of SNAP-23 protein is reduced by half in *Snap23* ^fl/wt^ and *Snap23* ^Δ/wt^ mice. (**A**) *Snap23* ^fl/wt^ heterozygous mice were mated and twelve two-week old pups from this mating were genotyped and analyzed for SNAP-23 protein expression. Genotyping of tail DNA was performed using PCR Primer set 2 from tail DNA. A single small PCR fragment (266 bp) is present in *Snap23* ^wt/wt^ pups, and double fragments (266 and 400 bp) is present in *Snap23* ^fl/wt^ pups. No homozygous *Snap23* ^fl/fl^ pups were obtained when more than 50 pups were analyzed from *Snap23* ^fl/wt^ heterozygous matings. For immunoblot analysis, whole brain was solubilized in modified RIPA lysis buffer and protein levels were analyzed by immunoblotting (WB) as indicated antibodies. (**B**) *Snap23* ^Δ/wt^ heterozygous mice were mated and pups from this mating were genotyped and their brains were analyzed for expression of SNAP-23 and other SNARE proteins. Genotyping of tail DNA was performed using PCR Primer set 2. A single small PCR fragment (266 bp) is present in *Snap23* ^wt/wt^ pups, and double fragments (266 and 492 bp) is present in *Snap23* ^Δ/wt^ pups. No homozygous *Snap23* ^Δ/Δ^ pups were ever obtained from *Snap23* ^Δ/wt^ heterozygous matings. Whole brains were solubilized and analyzed by immunoblotting (WB) using the indicated antibodies.

Brains from isolated from *Snap23* ^fl/wt^ mouse pups expressed only half as much SNAP-23 protein as *Snap23* ^wt/wt^ spleen cells, demonstrating that expression of SNAP-23 from the floxed allele was defective (Figure 3A). Identical results were observed when analyzing SNAP-23 expression in mast cells and spleen from *Snap23* ^fl/wt^ mice. These data are consistent with the idea that the loxP sites adjacent to the *Snap23* exon 2 splice donor/acceptor sites interfered with *Snap23* expression, a situation observed also in other mouse models ((15) and L.T., unpublished observations). Unlike their male counterparts, female EIIa-Cre trasgenic mice completely excise sequences between loxP sites [18], [19], and breeding these mice with male *Snap23* ^fl/wt^ mice resulted in the generation of S*nap23* ^Δ/wt^ mice. As expected, expression of SNAP-23 from the exon 2-deleted allele (*Snap23* ^Δ/wt)^ was also defective in the brains of *Snap23* ^Δ/wt^ mouse pups and densitometry confirmed that SNAP-23 expression was only half that observed in *Snap23* ^wt/wt^ littermates (Figure 3B). Expression of other SNARE proteins, including syntaxin 1a, syntaxin 3, syntaxin 13, SNAP-25, and VAMP2 was not altered in *Snap23* ^Δ/wt^ heterozygous mice.

### Deletion of Snap23 leads to early embryonic lethality

To generate homozygous *Snap23*-floxed mice, *Snap23* ^fl/wt^ mice were mated with each other. Genotyping of tail DNA from nearly 100 live pups failed to reveal any homozygous *Snap23* ^fl/fl^ mice, strongly suggesting that the *Snap23* ^fl^ allele was not expressed and that deletion of *Snap23* resulted in lethality. Instead of characterizing Snap23^fl/wt^ mice further, we set out to investigate the effects of *Snap23* deletion using exon 2-deleted *Snap23* ^Δ/wt^ mice. To generate *Snap23*-deficient mice, *Snap23* ^Δ/wt^ mice were mated with each other. As expected (based on our analysis of *Snap23* ^fl/wt^ mice), *Snap23* ^Δ/Δ^ pups were never obtained from adult heterozygous matings after more than 50 live births (data not shown). Genotyping confirmed that approximately 2/3 of these offspring were *Snap23* ^Δ/wt^ and 1/3 were *Snap23* ^wt/wt^, demonstrating that deletion of *Snap23* leads to embryonic lethality.

To determine at what embryonic stage *Snap23*-deficient mice were dying we obtained embryos isolated from the timed-pregnant *Snap23* ^Δ/wt^ heterozygous matings. No *Snap23* ^Δ/Δ^ embryos were recovered from 48 embryos obtained from embryonic day 16.5 (E16.5), E12.5, E11.5, E9.5, or E7.5, with 35 heterozygous and 13 wild-type embryos isolated (Table 2). Immunoblot analysis confirmed that SNAP-23 protein expression from heterozygous embryos was reduced by half as compared to wild-type embryos (data not shown). These results suggest that *Snap23*-null mice are dying at an early, pre-implantation developmental stage.

**Table 2:** Genotype analysis from timed-pregnant matings of *Snap23* ^Δ/wt^ heterozygote mice.

Age of embryos	No. of dissected embryos	No. of WT embryos (+/+)	No. of Het embryos (+/−)	No. of Homozygote embryos (−/−)
				
E16.5	7	4	3	0
E12.5	8	2	6	0
E11.5	8	2	6	0
E9.5	14	3	11	0
E7.5	11	2	9	0
E3.5	27	11	11	5

Embryo age, number of dissected embryos, and *Snap23* genotype results are summarized as indicated.

**Figure 4:**
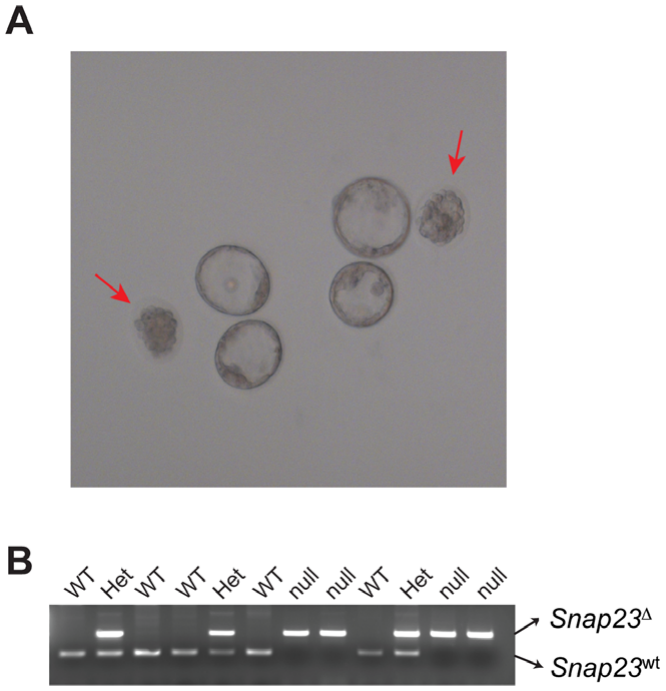
*Snap23* ^Δ/Δ^ blastocysts die prior to uterine implantation. (**A**) To evaluate the timing of embryonic lethality, embryos were collected from super-ovulated *Snap23* ^Δ/wt^ females mated with *Snap23* ^Δ/wt^ male mice by uterine flushing at E3.5. About 1/4 of the isolated blastocysts were morphologically abnormal and appeared to be degenerating; unlike sibling normal blastocysts they failed to develop any further during 24 hrs of culture (indicated by red arrows; see also Table 2). (**B**) Representative example of genotyping analysis revealing that abnormal blastocysts are homozygous for the *Snap23* deleted allele (*Snap23* ^Δ/Δ^). Genomic DNA was isolated from individual blastocysts (shown in panel (A)) following 24 hr in culture, and genotyping was conducted using primers genoE2 SS, genoE2 AS, and genoE3 rev. PCR products for the *Snap23* ^wt^ allele (266 bp) and for the *Snap23* ^Δ^ allele (492 bp) are indicated.

To determine if the *Snap23* null mutation is lethal before uterine implantation, blastocyst stage embryos were recovered by flushing from the uterus of pregnant mice at day E3.5. We noted that 5 among a total of 27 blastocysts isolated using this procedure appeared grossly abnormal and had not expanded properly, suggesting that these blastocysts were dying, and unlike the normal blastocysts, they failed to develop any further after 24 hrs of culture (arrows in Figure 4A). Genomic DNA was isolated from each blastocysts and genomic PCR was performed. Each of five small, degenerating blastocysts showed a *Snap23* ^Δ/Δ^ genotype (e.g. Figure 4B), whereas the morphologically normal embryos included 11 *SNAP23* ^Δ/wt^ heterozygotes and 11 *SNAP23* ^wt/wt^ wild-type embryos (Table 2). These results indicate that *Snap23* null embryos die prior to blastocyst implantation on the uterine wall.

SNAP-23 is a ubiquitously-expressed homolog of SNAP25 and has been shown to regulate membrane to membrane fusion process. Although we originally set-out to use Cre-mediated excision to conditionally delete *Snap23*, immunoblot analysis revealed that SNAP-23 protein expression in *Snap23* ^fl/wt^ mice was defective. Furthermore, breeding of *Snap23* ^fl/wt^ mice failed to generate homozygous *Snap23* ^fl/fl^ pups, a finding that is consistent with the failure of the floxed allele to express and the requirement for *Snap23* expression for embryo viability. We therefore chose to generate *Snap23* knockout mice using a conventional homologous recombination technique. Our data clearly demonstrated that *Snap23* homozygous knockout mice die prior to uterine implantation. Several knockout mice lacking various components of the SNARE machinery have been reported, and many of these did no show any gross abnormalities despite the fact that this class of proteins molecules is thought to be critical for essential biological process such as membrane protein recycling and protein secretion. The lack of a dramatic phenotype may be due to compensatory mechanisms by co-expressed SNARE isoforms playing a similar role (genetic redundancy) or in some cases because the roles of these proteins are confined to regulated exocytosis rather than constitutive protein trafficking events. For example, mice lacking VAMP3 [20], Synaptotagmin 4 [21], or HPC-1/Syntaxin 1A [22] are viable and appear to grow normally. In addition, SNAP-25, VAMP2, and munc-18 null mice are born live but die immediately after birth due to respiratory failure [14], [23], [24]. Synaptotagmin I null mice die within 48 h of birth [25]. VAMP8-null mice develop normally in our colony [26], however other groups have reported that VAMP8-deficient mice exhibit growth retardation and some perinatal lethality [27]. Compared to other known null-mutants of SNARE components, the early lethality of SNAP-23 null mice represents the essential role of SNAP-23 in SNARE-mediated vesicle-membrane fusion. While it is not possible to definitively attribute the early embryonic lethality observed here to defects in secretion, it should be noted that the yeast homolog of *Snap23*/*Snap25* (*SEC9*) is essential for cell viability [28]. In addition, our studies in neurons from *Snap23* heterozygous mice showed that they do indeed have defects in neurotransmitter receptor transport [13]. It is also interesting that syntaxin 4 is one of the major SNAP-23-binding proteins and syntaxin 4-deficient embryos also die before E7.5 [29], suggesting that SNAP-23/syntaxin 4 complexes are essential for a protein trafficking events in embryogenesis.

In conclusion, we generated *Snap23* knockout mice and demonstrated that *Snap23* deletion is lethal. Specifically, we showed that *Snap23*-null embryos die prior to implantation in the uterus. These data indicate that SNAP-23 plays a unique and essential role as a membrane fusion protein that is essential for cell viability.

## Materials and Methods

### Animals and antibodies

The use and care of animals used in this study followed the guidelines of the NIH Animal Research Advisory Committee. C57BL/6 mice were obtained from NCI-Frederick (Frederick, MD) and EIIa-Cre mice were obtained from The Jackson Laboratories (Bar Harbor, ME). All protocols were approved by the National Cancer Institute-Center for Cancer Research Animal Care and Use Committee (protocol numbers EIB-076 and EIB-094). Rabbit SNAP-23 antibody is described in a previous publication [13]. Syntaxin 1a (HPC 1; Wako chemicals), syntaxin 3 (Alomone labs), syntaxin 13 (15G2, Abcam), VAMP-2 (Cl 69.1; Synaptic Systems), α-tubulin (Sigma) antibodies were purchased from commercial sources as indicated.

### Generation of *Snap23* targeted mice

The Neo(+) *Snap23* exon 2 targeting allele was constructed from a BAC clone derived from a 129/SvJ mouse genomic library [16] by flanking exon 2 of the mouse *Snap23* gene with loxP sites. The targeting vector was constructed as follows: a 2 kb genomic DNA fragment upstream of *Snap23* exon 2 was obtained as a short arm by PCR using primers SA-4400SpeI and SA-6400SalI via SpeI/SalI restriction sites. All oligonucleotide primer sequences are described in Table 1. A neomycin resistance cassette (Neo) driven by the PGK promoter (in 3′ to 5′ direction) flanked by loxP sequences was cloned together with a short arm into SpeI/XbaI-digested pBluescript vector (pBS) (short arm-loxP-Neo-loxP). A genomic fragment harboring 278 bp upstream and 516 bp downstream of exon 2 was obtained by PCR using primers E2-6400SpeI and E2-7308KpnI via SpeI/KpnI restriction sites, which was further cloned into KpnI/XbaI-digested pBS, and another loxP was added on the 3′ end of exon 2 fragment (exon 2-loxP). The short arm-loxP-Neo-loxP fragment was further inserted into SpeI-digested exon 2-loxP in pBS (short arm-loxP-Neo-loxP-exon 2-loxP). A 4.9 kb long arm genomic DNA fragment was directly obtained from a BAC clone by EcoRI digestion. The thymidine kinase (HSV-tk) gene was cloned outside of the long arm fragment into ClaI/SalI-digested pBS (long arm-HSV-tk) for double selection. Finally, a SpeI linker was generated on the 3′ end of a short arm-loxP-Neo-loxP-exon 2-loxP fragment, which was further cloned into a long arm-HSV-tk in pBS by SpeI digestion. The nucleotide sequences of exon 2, loxP, PGK-Neo, and parts of short arm and long arm were verified by DNA sequencing. The targeting vector was linearized by NotI digestion and electroporated into the CJ7 embryonic stem (ES) cells as previously described [30]. The ES colonies were selected in the presence of G418/gancyclovir and analyzed for homologous recombination by Southern blot analysis using both 5′ and 3′ probes that were outside of the targeting vector. ES clones harboring a *Snap23* targeted allele were injected into blastocysts of the C57BL/6 mouse strain to generate germ-line chimeric offspring [31]. Offspring bearing the targeted *Snap23* allele were backcrossed more than four generations onto the C57BL/6 background before use to remove potential ES cell mutations not linked to the targeted allele.

### DNA analysis

ES cells, tissues, or mouse tails were lysed in 500 µl of DNA lysis buffer containing 40 mM Tris-HCl, pH 7.6, 200 mM NaCl, 20 mM EDTA, 0.5% sodium dodecyl sulfate (SDS), and 60 µg/ml proteinase K (Sigma-Aldrich) at 56°C overnight. Genomic DNA was extracted using phenol-chloroform followed by ethanol precipitation. The purified DNA was digested with EcoRI or ClaI/KpnI and analyzed by standard Southern blotting. All restriction enzymes were purchased from New England Biolabs. DNA templates for probe were amplified from a BAC DNA by PCR using primers E2-5probeSS and E2-5probeAS for 5′ probe, E2-3probeSS and E2-3probeAS for 3′ probe, and then purified from agarose gels. For PCR analysis, genomic DNA was obtained either by phenol-chloroform extraction or Extract-N-Amp Tissue PCR Kits (Sigma-Aldrich) according to the manufacturer's instructions. For the isolation of genomic DNA from blastocysts, QIAamp DNA Micro Kit (Qiagen) was used following the manufacturer's guidance. The sequence information of oligonucleotide primers used for PCR and the expected size of products are described in Table 1. PCR cycle consisted of 3 min at 94°C, then 36 cycles of 40 s at 94°C, 40 s at 58°C, 40 s at 72°C, and then 7 min at 72°C.

### Western blotting

Mouse whole brain was homogenized with a glass dounce homogenizer in modified RIPA buffer (50 mM Tris-HCl, pH 7.5, 150 mM NaCl, 2 mM EDTA, 1% NP40, 1% Triton X-100, 0.1% SDS, 0.5% sodium deoxycholate) containing EDTA-free complete protease inhibitor (Roche). The lysates were incubated for 30 min on ice and centrifuged at 20,000 *g* for 20 min. The supernatants were collected, resolved by SDS-PAGE, transferred to PVDF membranes, and analyzed by immunoblotting with the relevant antibodies as indicated. Bound antibodies were revealed using Western Lightening Chemiluminescence Reagent Plus (Perkin Elmer LifeSciences, Inc., Boston, MA).

### Isolation of blastocyst stage embryos

Four week-old *Snap23* ^Δ/wt^ females were induced to superovulate with sequential injections of FSH and HCG and bred with *Snap23* ^Δ/wt^ males using standard protocols [32]. The plug was examined the following day (noon considered as E0.5) and blastocysts were recovered at E3.5 by flushing the uterus. Collected blastocysts were maintained in culture for one day in M16 medium to reduce contamination by maternal tissues, and then lysed to purify genomic DNA for analysis.
